# SMART accelerates rate of cognitive gains in service members with mTBI and PTSD

**DOI:** 10.3389/fnhum.2025.1542422

**Published:** 2025-05-09

**Authors:** Erin Venza, Jeffrey S. Spence, Jennifer Zientz, Cole Devlin, Jason Bailie, Andrew Darr, Ida Babakhanyan, Sandra Bond Chapman

**Affiliations:** ^1^Center for BrainHealth, Behavioral and Brain Sciences, The University of Texas at Dallas, Dallas, TX, United States; ^2^Traumatic Brain Injury Center of Excellence (TBICoE), Silver Spring, MD, United States; ^3^General Dynamics Information Technology, Silver Spring, MD, United States; ^4^Naval Hospital Camp Pendleton, Camp Pendleton, CA, United States

**Keywords:** brain training, cognitive training, intervention, military, TBI, PTSD, executive function, randomized control trial

## Abstract

**Introduction:**

This randomized clinical trial (RCT) compared the efficacy of two cognitive rehabilitation (CR) protocols—Strategic Memory Advanced Reasoning Training (SMART) and Study of Cognitive Rehabilitation Effectiveness (SCORE)—in improving higher-order cognitive functions among active-duty service members (ADSMs) with mild traumatic brain injury (mTBI) and varying levels of post-traumatic stress disorder (PTSD) symptoms. The study also examined the relationship between PTSD symptom severity and cognitive outcomes.

**Methods:**

A total of 148 ADSMs with mTBI and persistent cognitive complaints were randomized to receive either SMART (20 h over 4 weeks) or SCORE (60 h over 6 weeks). High-level cognitive abilities were assessed with the Test of Strategic Learning and the Visual Selective Learning Task, and PTSD symptoms were measured using the PTSD Checklist (PCL-M). PTSD symptoms were accounted for as a covariate in all analyses. Outcomes were measured at baseline, post-treatment, and at 3-month follow-up.

**Results:**

Both SMART and SCORE groups showed significant improvements in complex memory and strategic learning, with no between-group differences in overall cognitive gains. Notably, SMART participants achieved these outcomes in one-third of the treatment hours. SMART also demonstrated greater immediate gains in fluency of high-level interpretations compared to SCORE (*p* = 0.04), reflecting enhanced possibility thinking. PTSD symptom severity was negatively correlated with performance on cognitive measures; however, the cognitive gains were comparable regardless of baseline PTSD symptoms.

**Discussion:**

SMART is an efficient and effective CR protocol for improving higher-order cognitive abilities in ADSMs with mTBI, achieving comparable outcomes to SCORE in 60% fewer treatment hours. Also of note, training-based cognitive gains were consistent across PTSD severity levels, suggesting CR is a potentially reliable tool for populations with mTBI plus concomitant PTSD. By promoting rapid cognitive improvement and adaptability, this study supports the potential for SMART to enhance the operational readiness of warfighters. Future research should explore hybrid delivery models and integration with PTSD-focused interventions to optimize accessibility and outcomes.

## 1 Introduction

In today’s increasingly complex and unpredictable global environment, military readiness depends on strengthening cognitive capacity, such as acuity, adaptability, and focus. Effort is urgently needed to complement a long-standing prioritization of physical strength and agility. Reliable metrics and interventions are critical to optimizing warfighter cognitive fitness. Mild traumatic brain injury (mTBI) is a significant health concern for active-duty service members (ADSMs) (Department of Defense, 2024). Cognitive impacts of mTBI are well-documented, affecting areas such as memory (Levin et al., 2012), complex attention and working memory (Vanderploeg et al., 2005). Whereas these deficits are most pronounced in the initial months after injury, functional deficits can persist for months or even years post-injury (Nelson et al., 2023). Furthermore, ADSMs with mTBI are at heightened risk of comorbid post-traumatic stress disorder (PTSD) which can further exacerbate cognitive vulnerabilities (Iljazi et al., 2020; Mattson et al., 2019). Our military forces require effective and efficient measures and interventions to continually perform at their optimal level and to restore functionality as rapidly as possible.

Effective cognitive rehabilitation (CR) protocols for ADSMs with mTBI are limited, with few well-designed studies in this population. The majority of studies have focused on *bottom-up* CR approaches, targeting foundational skills, such as memory or attention, through repetitive practice with the aim of rebuilding more complex abilities over time (Austin et al., 2024). One study examined a 10-week compensatory training (versus control group) in veterans with a history of mTBI and found improvements in domains of attention, learning, and executive functioning (Storzbach et al., 2017) The Study of Cognitive Rehabilitation Effectiveness (SCORE), compared three CR groups—a clinician-led CR, clinician-led CR plus cognitive-behavioral therapy (CBT), and computerized CR—against a psychoeducation group over 6 weeks and observed more modest gains (Cooper et al., 2017). Outcome measures included the Paced Auditory Serial Addition Test (PASAT) for attention and processing speed, the Global Distress Index for psychological distress, and the Key Behaviors Change Inventory (KBCI) for cognitive and behavioral deficits. While all groups showed significant improvements in attention and processing speed, no significant between-group differences were observed. Clinician-led CR groups demonstrated greater improvements in functional cognitive and behavioral abilities than psychoeducation or computerized CR, but these gains occurred in fewer than 25% of participants. Bottom-up approaches may have limited utility for enhancing higher-order cognitive abilities critical for active duty demands. Furthermore, the time-intensive nature of these research protocols make them impractical for clinical delivery, underscoring the need for more efficient interventions.

Recognizing the unfeasible duration and limited generalizability of bottom-up approaches to higher-order cognitive demands, researchers have explored top-down strategies as a more effective pathway for improving executive function and problem-solving (Chen and D’Esposito, 2010; Vas A. et al., 2016; Vas et al., 2011). These approaches emphasize metacognitive skills that promote self-agency and flexible thinking. One evidence-based intervention that addresses higher-order cognitive abilities is Strategic Memory Advanced Reasoning Tactics (SMART). SMART teaches meta-cognitive strategies that can be applied to all areas of life, from military operations to areas of personal relevance (relationships, purpose, quality of life, etc.) (Vas et al., 2011; Young et al., 2021). SMART strategies are grouped into three core principles: strategic attention, integrated reasoning, and innovation. Strategic Attention focuses on single-tasking, taking brain breaks regularly throughout the day, and the ability to focus on key elements while filtering less important information from the constant flow of massive data input. Integrated Reasoning requires the ability to take in large amounts of information and quickly distill it into its deeper-level meaning, promoting agility of reasoning and stability of decision making. Innovation targets expanding generative capabilities to build flexibility in deciphering a variety of relevant solutions, proactively generating possible next steps in case of failure, and consideration of issues from a variety of different perspectives and outcomes.

SMART is effective across a range of healthy and clinical populations, including individuals with TBI and those experiencing psychological distress and depression. Extant research on SMART reveals gains on single dimensions of memory and executive function (e.g., inhibition, switching, and verbal fluency) (Anand et al., 2011; Vas et al., 2011) as well as gains on measures of complex executive functions such as (1) strategic thinking (clearing the clutter and strategically finding the essential) (Young et al., 2021), (2) complex memory, reinforcing stability of new learning, (3) abstracting the gist (extracting deeper level meanings) (Chapman et al., 2015; Vas A. et al., 2016; Venza et al., 2016), and (4) innovating new ways to interpret complex input (Chapman et al., 2017; Young et al., 2021). In sum, SMART aims to strengthen self-agency by training individuals to exert cognitive control and intentionally direct mental energy to meet task demands. This is achieved through top-down information processing strategies that engage a broad network of higher-order cognitive domains.

SMART has demonstrated not only its ability to enhance complex executive functions but also its capacity to drive neuroplastic functional and structural changes in brain networks associated with higher order cognitive functions. A NIH-supported RCT demonstrated that SMART can mitigate brain losses associated with aging through enhanced neural plasticity, evidenced by increased cerebral blood flow, improved functional connectivity across brain networks, and enhanced white matter integrity (Chapman et al., 2015). In prior TBI research, SMART improved cognitive control, executive function, and psychological health, supported by neuroimaging evidence of enhanced network connectivity (Han et al., 2018), functional modularity (Han et al., 2020), cortical thickness, and neural efficiency (Han et al., 2017). Collectively, these findings position SMART as an effective intervention to promote brain health manifested through cognitive gains through targeted, top-down strategies that capitalize on neural adaptability.

The effectiveness of SMART with ADSM’s with mTBI is not well understood, as prior studies have focused on veteran populations and/or lacked a CR comparison group (Samuelson et al., 2020; Young et al., 2021). To address this void, a recently DOD-funded RCT compared the efficacy of SMART versus SCORE in enhancing cognitive function among ADSMs with mTBI and varying degrees of PTSD symptoms was conducted (Babakhanyan et al., 2020). Both groups showed significant cognitive and functional improvements from baseline to 3-month follow-up, with no between-group differences in outcomes (Darr et al., 2025). Notably, the SMART group accomplished the same degree of improvements in a third of the time of the SCORE group, highlighting its potential for efficient treatment delivery. This efficiency aligns with the military’s goal to expedite return-to-duty while minimizing clinical care demands.

One key factor in evaluating treatment effectiveness in mTBI is the contribution of concomitant PTSD. Individuals with PTSD also demonstrate cognitive deficits in frontally-mediated cognitive domains of attention, strategic memory, and executive function (Scott et al., 2015), and a recent imaging study noted similar abnormal connectivity patterns in individuals with mTBI and/or PTSD (Klimova et al., 2023). Building on this evidence, a recent meta-analysis reported that PTSD-related symptoms were negatively associated with cognitive performance in individuals with mTBI, suggesting that higher PTSD symptoms were associated with lower cognitive performance (Uiterwijk et al., 2022). These results highlight the need to account for PTSD symptoms when assessing CR-related cognitive outcomes in response to intervention protocols.

The current research examined the efficacy of SMART versus SCORE among ADSMs with mTBI and varying degrees of PTSD symptoms on novel outcomes of higher-order cognitive abilities. This current study advances the prior report (Darr et al., 2025) by accounting for PTSD symptoms in the model to better understand the impact of PTSD symptoms on CR training gains as measured by higher order cognitive outcome measures. Based on prior research showing higher levels of PTSD may be associated with lower cognitive performance (Uiterwijk et al., 2022), we predicted that more severe PTSD would be associated with lower performance on the novel measures of higher order executive functions. As mTBI and PTSD often coexist, if one remains untreated, it can hinder the progress of treating the other unless a treatment perhaps benefits both. To better understand this relationship, we have accounted for PTSD symptoms in our assessment of CR outcomes.

## 2 Materials and methods

### 2.1 Participants

A total of 148 ADSMs were recruited onsite at a military treatment facility. Participants were all active duty SMs with a history of at least one mTBI based on the DoD diagnostic criteria (VA/DoD, 2016). Exclusionary criteria included: (i) neurological diagnoses other than mTBI including multiple sclerosis, cerebral vascular accident, brain tumor, neurodegenerative disease, and neuro-motor disorder; (ii) mTBI history within the last 3 months or any history of moderate or severe TBI; (iii) current substance use disorder, or (iv) active suicidal or homicidal ideations. There was no limitation based on age, race, or ethnicity. All participants included had a non-penetrating head injury and reported either loss of consciousness of less than 30 min or being dazed at the time of injury as measured on the Ohio State University (OSU) TBI Identification Method (Corrigan and Bogner, 2007). Both groups (SCORE and SMART) had similar demographic, injury, and PTSD characteristics, with minor differences in education, repeated TBI rates, and age at first TBI. The SMART group had slightly higher average age, higher rates of repeated TBIs, and a slightly older age at first TBI compared to the SCORE group (see Table 1).

**Table 1:** Baseline demographic and characteristics.

Characteristics	Overall	SCORE (*n* = 68)	SMART (*n* = 80)
Age (M, SD)	33.80, 8.07	32.22, 8.44	35.13, 7.53
Male gender (*n*, %)	133, 90%	59, 87%	74, 93%
White race (*n*, %)	113, 76%	51, 75%	62, 78%
Hispanic ethnicity (*n*, %)	40, 27%	18, 26%	22, 28%
High school degree or some college (*n*, %)	110, 74%	56, 82%	54, 68%
Mild TBI (*n*, %)	148, 100%	68, 100%	80, 100%
Repeated TBI (*n*, %)	126, 85%	56, 82%	70, 88%
Age at first TBI (M, SD)	17.54, 7.13	16.48, 6.82	18.44, 7.31
Mean PCL score (SD)	47.96, 17.02	47.28, 17.42	48.49, 16.77

### 2.2 Measures

Outcome measures were administered before training, within 2 weeks post-training, and 3 months later. The Post-Traumatic Stress Disorder Checklist (PCL-M) was used to assess PTSD symptoms (Weathers et al., 1993). This 17-item self-report is specifically designed for use with ADSMs and veterans. The PCL-M yields a total symptom severity score ranging from 17 to 85, where a higher score indicates more symptoms.

The Test of Strategic Learning (TOSL), developed at the Center for BrainHealth at The University of Texas at Dallas, was designed to evaluate complex information-processing abilities that are essential to effective and agile synthesis of incoming information. Participants read an approximate 600-word text and then performed three tasks: (1) summarize the text to capture the high-level ideas conveyed (abstraction and synthesis), (2) generate multiple interpretations or “take-home messages” (fluency of higher-order interpretations), and (3) recall key details from the text (complex memory). Unlike other text assessments of complex memory (Lambez and Vakil, 2020), the TOSL’s structure allows researchers to examine two levels of information processing, i.e., both abstracted gist-level and complex memory performance. The TOSL has a manualized scoring system that tallies abstracted ideas, high-level synthesis and interpretations and accurate key points of detail-level responses. Alternate versions of TOSL were administered at each timepoint to minimize practice effects. The TOSL has acceptable test–retest reliability and is a validated measure in TBI with good sensitivity (84.7%) and specificity (71.1%) (Vas A. K. et al., 2016).

The Visual Selective Learning (VSL) task assesses the ability to strategically manage, prioritize, and remember information in real time. Successful performance requires a goal-oriented strategy, which calls upon both cognitive control, executive functions and memory (Castel et al., 2011; Miric, 2018). Individuals are presented with 3 subsequent lists of single words (20 words/list). The first list is treated as a practice round, and final scores are based on performance on the second and third lists. Based on certain criteria, half of the words yield a high-point value (10 point) and the other half yield a low-point value (1 point). Individuals are instructed to remember as much as they can, with the goal being to earn as many points as possible. Alternate versions of the VSL task were administered at each timepoint to minimize practice effects.

### 2.3 Interventions

Two speech-language pathologists were trained to deliver both interventions, and they alternated between the two based on the randomization protocol.

The SCORE protocol is a 6-week, 60-h program combining compensatory strategy training and computer-based Attention Processing Training (APT-3) (see Tables 2, 3). Weekly sessions include 5 individual, 2 group, and 3 computer-based sessions. Compensatory strategies address *attention* (sustained and divided), *planning* (problem-solving, sequencing, time management), and *memory* (association, rehearsal, visual/tech-based cues) (Cooper et al., 2017). Performance is improved through repetition, errorless learning, and gradually increasing task stimuli and complexity in a structured systematic approach. Additionally, participants work with trainer to set, track, and work toward personalized, functional goals through the Goal Attainment Scaling (GAS) method. These goals are typically related to the specific cognitive domains of focus (attention, memory, etc.), but they can also be related to emotional or functional domains of daily life.

**Table 2:** Intervention delivery.

Intervention schedule and delivery	
	SCORE delivery
Weeks 1–6	5 days/week: 1-h individual compensatory training 3 days/week: 1-h group compensatory strategy training 2 days/week: 1-h individual APT
	SMART delivery
Week 1	5 days/week: 1-h group strategy instruction
Weeks 2, 3, 4	3 days/week: 1-h group strategy implementation 2 days/week: 1-h individual goal-focused sessions

**Table 3:** Intervention concepts and examples.

Core concepts	Examples
SCORE intervention	
Sustained attention	Practice tasks that require sustained attention to complete effectively (e.g., error-monitoring tasks, note-taking tasks, math problems, puzzles)
Alternating attention	Practice dual-task exercises (e.g., card sorting tasks)
Organization	Practice tasks requiring hypothetical problem solving or time management scenarios (e.g., medication management)
Memory	Practice memory techniques (e.g., repetition, visual imagery) and utilizing external aids to support recall
SMART intervention	
Strategic attention	Practice single-tasking to focus attention and filter out irrelevant distractions and input; Promote mental calm by deliberately disconnecting from technology and social distractions
Integrated reasoning	Extract synthesized ideas from complex information and apply to make real-time decisions and solve problems
Innovation	Reframe mistakes as multiple learning opportunities; Challenge the status quo by exploring an array of novel approaches and cultivating curiosity

The SMART protocol consisted of 5 h of training per week over 4 weeks (total 20 h). The first week focused on strategy instruction, with the core strategies being trained over 5 group sessions. Strategic attention strategies support mental energy, focus and productivity throughout the day. Integrated Reasoning strategies support effective information-processing abilities with daily contexts and complex information. Innovation strategies support mental flexibility, perspective taking, and solution generation to support possibility thinking. The remaining 3 weeks focused on strategy implementation with exercises and contexts relevant to participants’ military responsibilities and personal lives. During individual sessions, participants identified two goals they wanted to accomplish that would support their brain health (e.g., related to cognition, social connection, well-being, lifestyle, etc.). After identifying the specific goal, they outlined how they would implement the specific SMART strategies to make meaningful progress toward that goal. Individual sessions were focused on goal-setting support and aligning the strategies with personally meaningful outcomes. Participants received feedback from the trainer not only relative to performance on in-session group interactions regarding complex cognitive activities, but also regarding their responses on applied activities.

### 2.4 Statistical analysis

We implemented a linear mixed effects model in the R statistical computing language^1^ for each of five cognitive measures—three TOSL outcomes and two VSL trials. Each dependent variable was modeled as an effect due to training group (SCORE or SMART), time (Baseline, 3 months and 6 months post-training), the covariate PCL to account for variability due to the number of PTSD symptoms, and all two-way and three-way interactions involving these terms. Separate variance components were estimated for between-subject variability and within-subject variability over time, since measurements within subjects over time are positively correlated. In the absence of interactions with PCL, primary interest concerned mean temporal contrasts and whether these contrasts differed by training group, i.e., group by time interactions conditional on the average PCL score. Additionally, we tested the associations between PCL and each dependent variable. All tests of contrasts and of PCL associations were based on t-statistics without multiple testing adjustments.

## 3 Results

### 3.1 PTSD symptoms and cognitive outcomes

**Figure 1:**
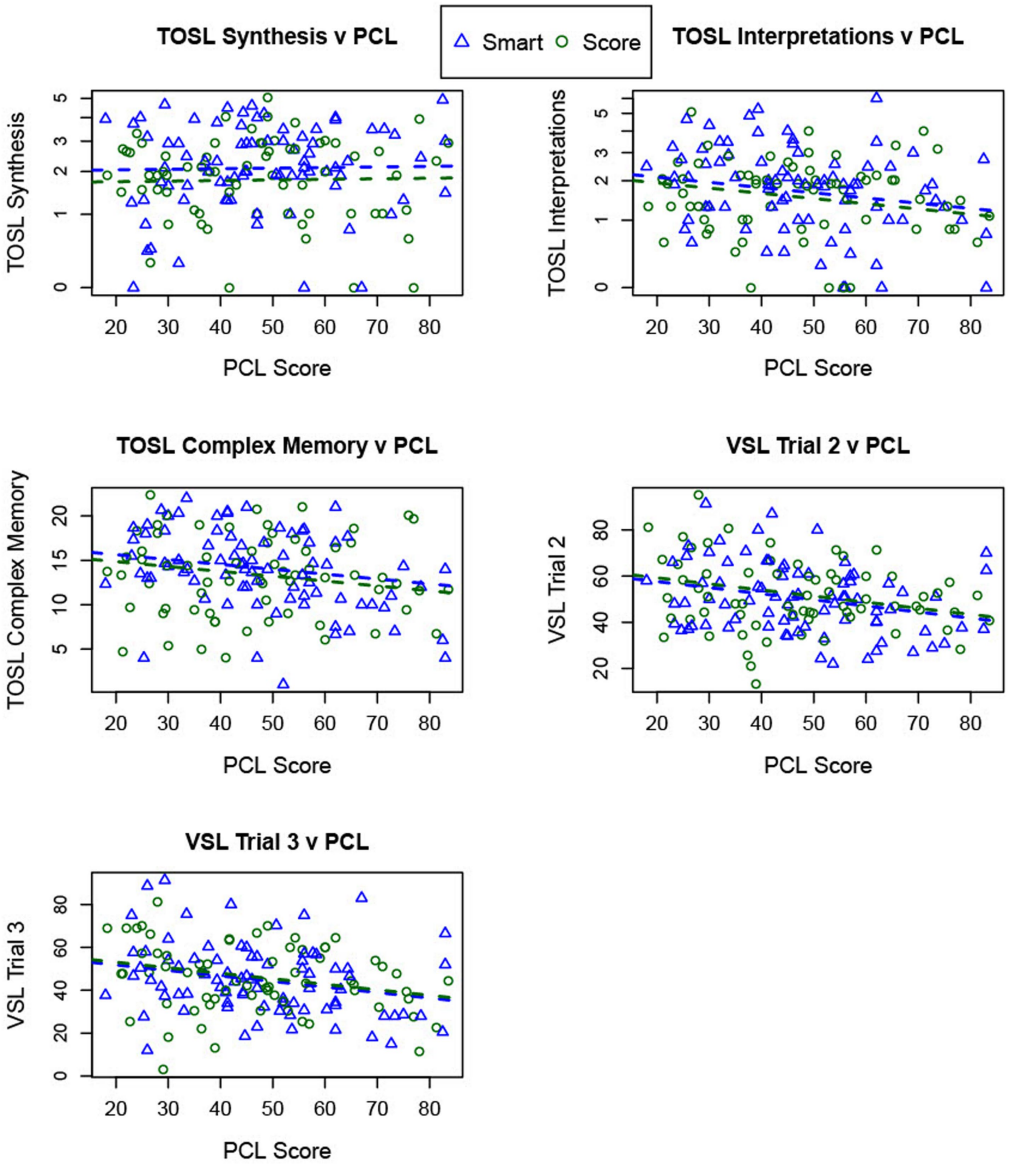
The figure depicts PTSD symptom severity and cognitive outcomes. Average PTSD symptoms (PCL score, x-axis) are plotted against each cognitive measure (y-axis) for both training groups (SCORE = green circle, SMART = blue triangle). Higher PCL scores predict lower performance on TOSL High-level Interpretations, TOSL Complex Memory, and both Visual Selective Learning (VSL) trials. No such relationship appears for TOSL Synthesis v PCL (upper left corner).

PCL scores did not have any significant interactions with training group and time, suggesting that any association of PCL with cognitive outcomes did not depend on what type of training was implemented or on average change scores over time. Averaged PCL scores had a significant negative association with four of the five cognitive measures, such that higher levels of PTSD symptoms were associated with lower scores on measures of (1) high-level interpretations (*p* ≤ 0.01, *r* = −0.25), (2) complex memory (*p* ≤ 0.01, *r* = −0.23), and (3) strategic learning trial 2 (*p* ≤ 0.001, *r* = −0.28) and trial 3 (*p* ≤ 0.01, *r* = −0.27) (see Figure 1; Table 4). Given this relationship, we accounted for PTSD symptoms in our assessment of CR outcomes.

**Table 4:** Regression model summary.

Measure	Estimate	SE	t-statistic	*p*-value
Synthesis v PCL	0.000289	0.000871	0.331941	0.74
Interpretations v PCL	−0.00256	0.000896	−2.85713	<0.01**
Complex memory v PCL	−0.05744	0.020201	−2.84346	<0.01**
Strategic learning, trial 2 v PCL	−0.258874	0.07583	−3.41386	<0.001***
Strategic learning, trial 3 v PCL	−0.255168	0.076166	−3.35014	<0.01**

### 3.2 Mean changes over time and type of training conditional on average PCL score

**Figure 2:**
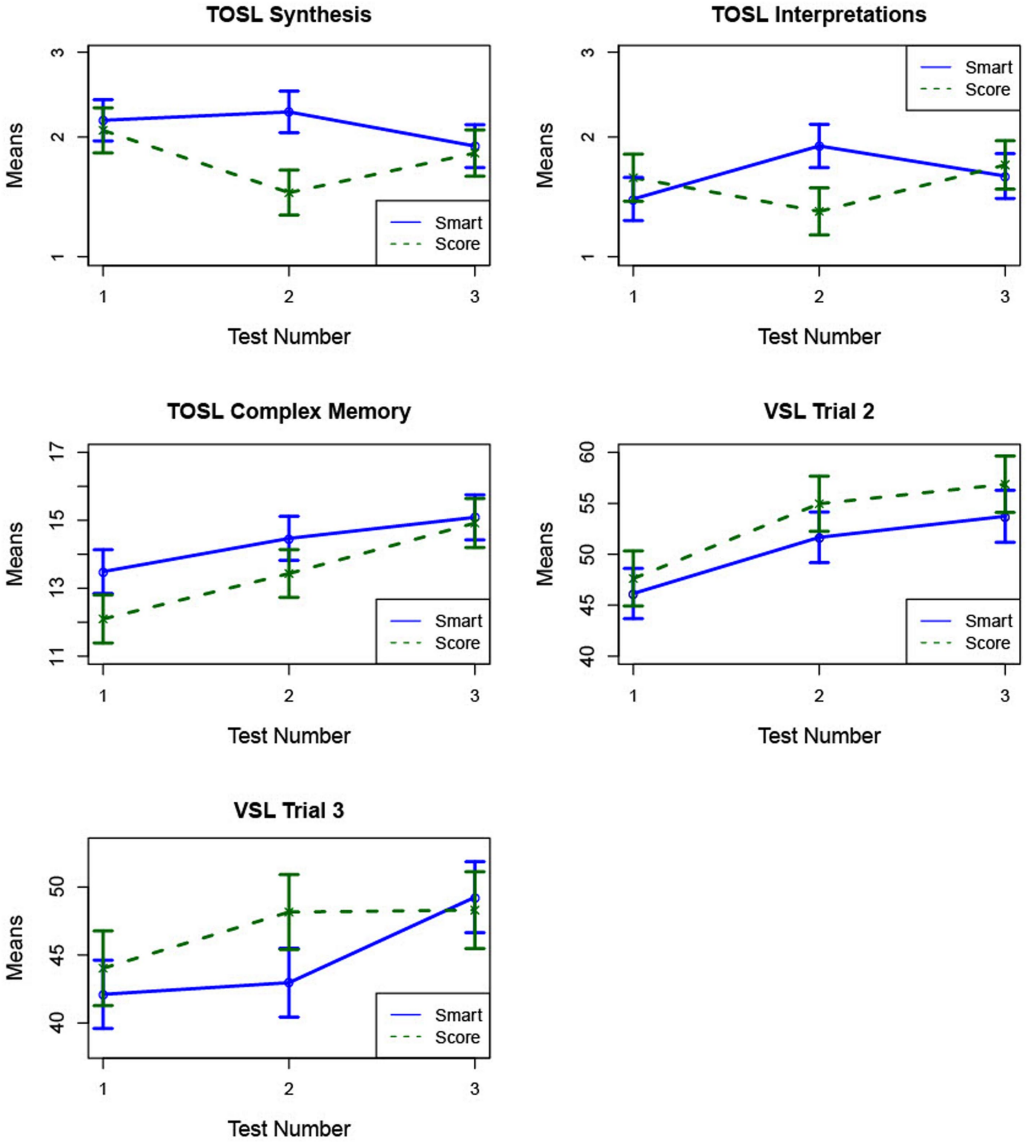
The figure demonstrates mean cognitive change (± SE) for SCORE and SMART from pre-training (1) to post-training (2) and 6-week follow-up (3). Group × Time interactions: SMART group improved more than the SCORE group from T1→T2 on TOSL Synthesis and TOSL High-level interpretations. Main effects of time: TOSL Complex Memory and VSL Trials 2 & 3 rise significantly from T1→T2 and T1→T3 for both groups.

High-level synthesis: The SCORE group showed a significant decrease from time 1 to time 2 (*p* = 0.03) but no significant change from time 1 to time 3 (*p* = 0.44). Although the SMART group showed no significant changes over time for high-level synthesis between time 1 and time 3 (*p* = 0.75), the SMART group had a higher mean change relative to the SCORE group between time 1 to time 2 (*p* = 0.07) (Figure 2; Table 5).

**Table 5:** Temporal contrasts and interactions with training group conditional on average PCL score.

	Contrasts and Interactions	Estimate	SE	t-statistic	*p*-value
High-level synthesis	SCORE T2-T1	−0.09	0.04	−2.17	0.03
	SCORE T3-T1	−0.03	0.04	−0.78	0.44
	SMART T2-T1	0.01	0.04	0.32	0.75
	SMART T3-T1	−0.04	0.04	−0.96	0.34
	SMART v SCORE T2-T1	0.10	0.06	1.81	0.07
	SMART v SCORE T3-T1	0.00	0.06	−0.08	0.94
High-level interpretations	SCORE T2-T1	−0.05	0.04	−1.11	0.27
	SCORE T3-T1	0.02	0.05	0.41	0.68
	SMART T2-T1	0.08	0.04	1.89	0.06
	SMART T3-T1	0.03	0.04	0.80	0.42
	SMART v SCORE T2-T1	0.13	0.06	2.10	0.04
	SMART v SCORE T3-T1	0.02	0.06	0.24	0.81
Complex memory	SCORE T2-T1	1.34	0.74	1.82	0.07
	SCORE T3-T1	2.82	0.75	3.76	<0.001
	SMART T2-T1	0.98	0.68	1.44	0.15
	SMART T3-T1	1.60	0.70	2.30	0.02
	SMART v SCORE T2-T1	−0.36	1.00	−0.36	0.72
	SMART v SCORE T3-T1	−1.23	1.02	−1.20	0.23
Strategic learning, trial 2	SCORE T2-T1	7.34	3.06	2.40	0.02
	SCORE T3-T1	9.25	3.12	2.97	<0.01
	SMART T2-T1	5.51	2.82	1.95	0.05
	SMART T3-T1	7.58	2.88	2.63	0.01
	SMART v SCORE T2-T1	−1.83	4.16	−0.44	0.66
	SMART v SCORE T3-T1	−1.67	4.25	−0.39	0.70
Strategic learning, trial 3	SCORE T2-T1	4.14	3.24	1.28	0.20
	SCORE T3-T1	4.28	3.30	1.30	0.20
	SMART T2-T1	0.86	2.98	0.29	0.77
	SMART T3-T1	7.15	3.05	2.35	0.02
	SMART v SCORE T2-T1	−3.28	4.40	−0.75	0.46
	SMART v SCORE T3-T1	2.87	4.49	0.64	0.52

High-level interpretations: The SCORE group did not demonstrate significant changes in fluency of high-level interpretations between time 1 and time 2 (*p* = 0.27), while the SMART group showed an increase (*p* = 0.06). Moreover, the mean increase for SMART was significantly larger than the mean change for the SCORE group (*p* = 0.04), with a medium effect size (*d* = 0.52), indicating better performance on high-level interpretations for SMART training relative to SCORE training.

Complex memory: Both groups showed improvements in memory for key text details following training. The SCORE group demonstrated an increase from time 1 to time 2 (*p* = 0.07) and a significant increase from time 1 to time 3 (*p* < 0.001). The SMART group showed a significant gain in memory from time 1 to time 3 (*p* = 0.02). These mean changes were not statistically different between the training groups.

Strategic learning, Trial 2: The SCORE group showed significant increases from time 1 to time 2 (*p* = 0.02) and from time 1 to time 3 (*p* < 0.01). The SMART group also showed improvement, with an increase from time 1 to time 2 (*p* = 0.05) and a significant increase from time 1 to time 3 (*p* = 0.01). These mean changes were not statistically different between the training groups.

Strategic learning, Trial 3: The SCORE group did not show significant changes across time points. Although the SMART group demonstrated a significant improvement from time 1 to time 3 (*p* = 0.02), these mean changes were not statistically different between the training groups.

### 3.3 Stability of PTSD symptomatology effects on cognitive outcomes over time

The negative associations between PTSD symptom severity (as measured by PCL scores) and cognitive outcomes at T1 were not significantly different from those observed at T2 or T3, indicating that the relationship between PTSD symptom severity and cognitive performance remains stable over time, regardless of the type of training intervention implemented (Table 6: PCL × time effect).

**Table 6:** Associations between PCL and cognitive outcomes over time.

	PCL × Time effect		Time effect	
	F-statistic (2,205)	*p*-value	F-statistic (2,205)	*p*-value
High-level synthesis	0.48	0.62	1.15	0.32
High-level interpretations	1.27	0.28	0.36	0.70
Complex memory	1.55	0.21	9.35	<0.001
Strategic learning, trial 2	2.46	0.09	8.73	<0.001
Strategic learning, trial 3	0.23	0.79	3.25	0.04

**Figure 3:**
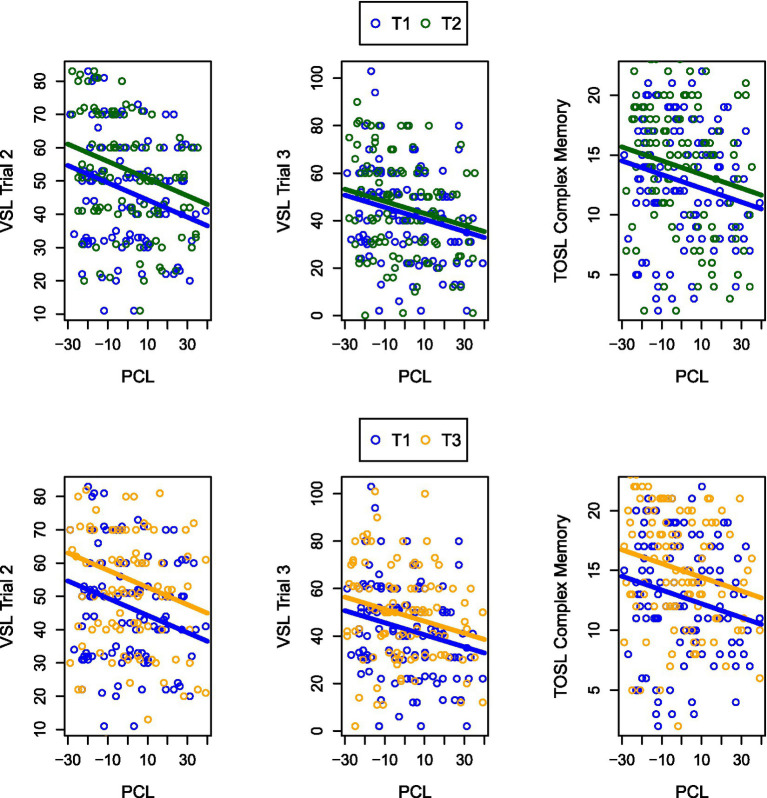
The figure illustrates cognitive gains across PTSD severity levels. Plots demonstrate cognitive gains for pooled SMART + SCORE data regardless of PTSD severity levels (PCL) for the Visual Selective Learning trials 2 & 3 and TOSL Complex Memory. Specifically, in the top three plots, the bottom bar shows baseline performance (T1) and the higher bar shows post-training performance (T2) for cognitive outcomes versus PCL severity. In the bottom three plots, the bottom bar indicates pre-training performance, and the upper bar is 6-week post training performance for cognitive outcomes versus PCL severity.

A significant main effect of time was observed for three of the outcome measures: Strategic Learning, Trial 2, Strategic Learning, Trial 3, and Complex Memory. Significant increases were observed from T1 to T2 and T1 to T3 for these measures (*d* = 0.44, *d* = 0.29, and *d* = 0.44, respectively), suggesting improvements in cognitive performance over time (see Figure 3; Table 6: Time effect). Importantly, these improvements were not conditional on PTSD symptom severity but rather comparable regardless of symptom severity. In contrast, no significant time effects were observed for High-level Synthesis or Interpretations. For the High-level synthesis and Interpretations, this lack of effect may be attributable to a group × time interaction at T2, and at T3, there was no significant effect detected.

## 4 Discussion

This RCT compared the efficacy of SMART versus SCORE on outcomes of complex cognition in ADSMs with mTBI and persistent cognitive complaints. SMART is a strategy-based training that has been shown to improve higher order executive functions in healthy and brain-injured populations (Chapman et al., 2015; Vas A. K. et al., 2016; Young et al., 2021). Although SMART is more novel to the DOD compared to SCORE, its efficacy in improving higher-order cognitive abilities for mTBI with persistent symptoms is promising given its shorter timeline for treatment. In this study, we examined the influence of PTSD symptomatology on cognitive outcomes and found that irrespective of self-reported PTSD symptoms there are cognitive gains noted following a manualized CR treatment protocol.

Four interesting findings emerged from this randomized clinical trial. First was the significant relationship between the novel higher-order top-down cognitive outcomes and PTSD-related symptoms. While it has long been suspected that co-occurring PTSD with TBI exacerbates cognitive sequelae, no known study has examined the relationship between mTBI and severity of PTSD on higher order complex cognitive measures. Previous research has reported an inverse relationship between PTSD and cognitive performance on traditional metrics of distinct specific measures of processing speed (Thompson et al., 2024), category fluency, verbal recall, and verbal recognition cognition (Cohen et al., 2013). This effect extends to critical cognitive skills such as strategic learning, information processing, and adaptive thinking fluidity—abilities essential for warfighters. Specifically, higher symptoms of PTSD in those with mTBI was associated with lower performance on measures of complex cognitive abilities—regardless of time point or training group. The relationship between PTSD and cognitive performance likely reflects overlapping disruptions in underlying frontal-lobe-mediated functions (Scott et al., 2015; Uiterwijk et al., 2022).

A second finding was that CR effectively enhanced cognitive capacity in ADSMs with mTBI, irrespective of comorbid PTSD symptom severity. This study underscores the adaptability and inclusivity of CR protocols that are clinician-led and incorporate meta-cognitive strategies and personal goal-setting components. Such CR approaches can address diverse cognitive needs, apply to a range of daily contexts, and foster cognitive readiness in ADSMs. Whereas prior studies also noted a reduction in PTSD symptoms post-SMART (Vas A. K. et al., 2016; Samuelson et al., 2020), the current study findings suggest a strengthening of cognitive systems across varying emotional states, making it a versatile and impactful tool for active-duty service members.

The third finding was a trend toward greater gains from SMART training versus SCORE, at least immediately after training (T2) on two measures of complex executive function and reasoning, i.e., high-level synthesis and high-level interpretations. Perhaps with continued or tapered training overtime, SMART could outpace SCORE longitudinally. As a reminder, we adjusted our model to account for PTSD symptomatology to carefully distinguish any training-induced cognitive changes between the SMART and SCORE groups. The SMART group showed a higher mean-level improvement relative to SCORE immediately following training on both measures. This immediate training improvement for the SMART groups suggests that gains were beginning to emerge. Moreover, our findings suggest that with continuous practice/training, SMART could also prove more effective than SCORE over time while still benefiting both military health systems and patients in terms of time and money.

One unique cognitive benefit of SMART was its impact on fluency in high-level interpretations, a generative skill we term “possibility thinking.” This skill aligns with multiple higher-order cognitive processes, such as reasoning, evaluating, and perspective-taking, to foster deep learning and problem-solving that extend far beyond simple factual memory (Gamino et al., 2010). Previous studies in TBI and military populations (Vas A. K. et al., 2016; Young et al., 2021) have demonstrated that SMART’s top-down strategies significantly enhance complex cognitive abilities, including “possibility thinking,” which are critical for decision-making and operational readiness in active-duty service members. Unlike SCORE’s reliance on repetitive compensatory strategies that target lower-order cognitive skills such as recall and rehearsal, SMART focuses on meta-cognitive strategies that promote strategic thinking and adaptability across diverse life contexts. Such an approach not only engages broader cognitive networks, including the Central Executive and Default Mode Networks (Chapman et al., 2017), but also fosters neural plasticity that supports sustained learning and adaptability.

The final, and perhaps most significant, finding relevant for ADSMs to return to duty was that SMART achieved comparable and even trending toward higher gains in one third the time spent in treatment (i.e., 20 h versus 60 h). Results suggest the SMART strategies are more readily adaptable to practice in daily life responsibilities. As such, SMART strategies may promote faster cognitive gains and transfer to complex tasks. The potential for faster cognitive improvement may be driven by the top-down strategic thinking tactics, which target cognitive control processes and engage multiple brain networks across the prefrontal cortex (Chen et al., 2006). In contrast, interventions focusing on lower-level or discrete cognitive abilities often require more extensive training to yield improvements. For instance, compensatory approaches have ranged from 18 to 24 h (Cooper et al., 2017; Storzbach et al., 2017) and a recent computerized training protocol in mTBI required 65 h of training over 13 weeks (Mahncke et al., 2021). In this study, SMART was delivered over 4 weeks to align with prior laboratory research, yet SMART has been successfully adapted and deployed in as little as 1 week with military personnel (Young et al., 2021). Understandably, shorter protocols are beneficial to ADSM’s, who have unpredictable schedules, and clinicians, who often accommodate last-minute scheduling changes.

This study had several limitations that should be addressed in future research. First, the absence of a non-treatment control group prevents us from determining the extent to which the observed improvements can be attributed to (i) CR only, (ii) the other interventions participants were also receiving as part of a multidisciplinary TBI treatment setting, or (iii) practice effects. However, based on prior studies that evaluated similar cognitive training protocols using waitlist control groups, we suggest that the improvements observed in our cohorts here are unlikely to be fully explained by practice effects alone (Chapman et al., 2015, 2017). Furthermore, in a single-arm SMART study, cognitive gains were associated with training completion and were minimal or absent among those who did not engage in the training (Chapman et al., 2021), reinforcing that practice effects alone cannot account for these improvements. Second, as the study was conducted with individuals with mTBI at a single military treatment facility, this limits the generalizability of the findings to individuals with moderate–severe TBI and/or other military contexts or civilian populations. Third, while the outcome measures targeted complex cognitive abilities, they did not include objective indicators of return-to-duty rates or enhanced mission readiness in real-world military settings. Lastly, when this study protocol was created, only the 60-h SCORE protocol was available, but a shorter 20-h version has been developed and is currently being validated. Future studies should examine whether shorter or more intensive versions of either protocol can produce comparable cognitive benefits in ADSMs and help establish the minimal effective dose.

Finally, technology-enhanced delivery models warrant future exploration. Hybrid approaches that integrate online tools with live clinicians could maintain the effectiveness of in-person trainings while making CR more accessible to service members, regardless of location. Until recently, online CR protocols in this population have been restricted to bottom-up approaches, as they are more easily scaled than top-down strategy-based trainings. A recent online research platform has the capability of (i) assessing holistic domains of brain health, (ii) delivering online SMART training, and (iii) offering one-on-one coaching to guide goal-direction application of SMART strategies in various research contexts (Chapman et al., 2021). Future studies should explore whether a hybrid approach in this population would yield comparable results to in-person studies.

Ultimately, the findings of this study advance the evidence that CRs offers a significant treatment option to enhance the cognitive performance of ADSMs with mTBI. Results also emphasize the interconnectedness of psychological health and cognitive health in this population. By equipping service members with the tools to recover higher-order cognitive abilities efficiently, interventions like SMART can significantly contribute to mission readiness, ensuring warfighters are prepared to meet the complex demands of their duties.

## Data Availability

The data analyzed in this study is subject to the following licenses/restrictions: The raw data supporting the conclusions of this article will be made available upon request. Requests to access these datasets should be directed to Erin Venza, erin.venza@utdallas.edu.

## Footnotes

^1^ http://r.project.org
